# Supplementary material for: Cervical cancer screening outcomes for HIV-positive women in the Lubombo and Manzini regions of Eswatini—Prevalence and predictors of a positive visual inspection with acetic acid (VIA) screen
Source: PLOS Glob Public Health. 2024 Apr 16;4(4):e0002760. doi: 10.1371/journal.pgph.0002760 (PMC11020862; doi:10.1371/journal.pgph.0002760)
Supplement: S1 Table — (DOCX) [file pgph.0002760.s001.docx]

SUPPLEMENTARY MATERIAL

S1 Table: Completed STROBE Statement

|  | **Item No.** | **Recommendation** | **Page  No.** | **Relevant text from manuscript** |
| --- | --- | --- | --- | --- |
| **Title and abstract** | 1 | (*a*) Indicate the study’s design with a commonly used term in the title or the abstract | 2 | Methods in abstract |
|  |  | (*b*) Provide in the abstract an informative and balanced summary of what was done and what was found | 2 | Abstract methods and results |
| **Introduction** | | | |  |
| Background/rationale | 2 | Explain the scientific background and rationale for the investigation being reported | 3, 4, 5 | This is described in the Introduction |
| Objectives | 3 | State specific objectives, including any prespecified hypotheses | 6 | In this study, we describe the cervical cancer screening outcomes, prevalence, and predictors of a positive cervical screen among HIV-positive women accessing services at selected health facilities in two regions of Eswatini. |
| **Methods** | | | |  |
| Study design | 4 | Present key elements of study design early in the paper | 6,7 | This study used a retrospective cohort design among HIV-positive women receiving care at select health facilities.in Eswatini. |
| Setting | 5 | Describe the setting, locations, and relevant dates, including periods of recruitment, exposure, follow-up, and data collection | 7, 8 | Study design, context, and study setting, participants, sample size and sampling. |
| Participants | 6 | (*a*) *Cohort study*—Give the eligibility criteria, and the sources and methods of selection of participants. Describe methods of follow-up  *Case-control study*—Give the eligibility criteria, and the sources and methods of case ascertainment and control selection. Give the rationale for the choice of cases and controls  *Cross-sectional study*—Give the eligibility criteria, and the sources and methods of selection of participants | 9 | Section under study participants, sample size and sampling. |
|  |  | (*b*) *Cohort study*—For matched studies, give matching criteria and number of exposed and unexposed  *Case-control study*—For matched studies, give matching criteria and the number of controls per case |  |  |
| Variables | 7 | Clearly define all outcomes, exposures, predictors, potential confounders, and effect modifiers. Give diagnostic criteria, if applicable | 9, 10 | Described under data sources and study variables; Statistical analysis |
| Data sources/ measurement | 8* | For each variable of interest, give sources of data and details of methods of assessment (measurement). Describe comparability of assessment methods if there is more than one group | 9 | Described under data sources and study variables |
| Bias | 9 | Describe any efforts to address potential sources of bias | 8, 9, 10 | We tried to limit selection bias by including clients from all health facilities in the Lubombo and Manzini regions that provide cervical cancer screening services. |
| Study size | 10 | Explain how the study size was arrived at | 9, 10 | Study participants, Sample size and sampling |

| Quantitative variables | 11 | Explain how quantitative variables were handled in the analyses. If applicable, describe which groupings were chosen and why | 9, 10 | Data sources and study variables |
| --- | --- | --- | --- | --- |
| Statistical methods | 12 | (*a*) Describe all statistical methods, including those used to control for confounding | 9, 10 | Statistical analysis |
|  |  | (*b*) Describe any methods used to examine subgroups and interactions | 9, 10 | Statistical analysis |
|  |  | (*c*) Explain how missing data were addressed | 9, 10 | Described under statistical analysis. Case-wise analysis was used. |
|  |  | (*d*) *Cohort study*—If applicable, explain how loss to follow-up was addressed  *Case-control study*—If applicable, explain how matching of cases and controls was addressed  *Cross-sectional study*—If applicable, describe analytical methods taking account of sampling strategy | 9, 10 | Loss to follow-up was not assessed |
|  |  | (*e*) Describe any sensitivity analyses |  | None conducted |
| **Results** | | | | |
| Participants | 13* | (a) Report numbers of individuals at each stage of study—eg numbers potentially eligible, examined for eligibility, confirmed eligible, included in the study, completing follow-up, and analysed | 11, 12 | Table 2 |
|  |  | (b) Give reasons for non-participation at each stage |  |  |
|  |  | (c) Consider use of a flow diagram |  | Not indicated |
| Descriptive data | 14* | (a) Give characteristics of study participants (eg demographic, clinical, social) and information on exposures and potential confounders | 11, 12 | Table 2 |
|  |  | (b) Indicate number of participants with missing data for each variable of interest |  | Not done |
|  |  | (c) *Cohort study*—Summarise follow-up time (eg, average and total amount) | 11, 12 | Table 2: Duration on ART treatment |
| Outcome data | 15* | *Cohort study*—Report numbers of outcome events or summary measures over time | 12 | This is reported in Table 3 |
|  |  | *Case-control study—*Report numbers in each exposure category, or summary measures of exposure |  |  |
|  |  | *Cross-sectional study—*Report numbers of outcome events or summary measures |  |  |
| Main results | 16 | (*a*) Give unadjusted estimates and, if applicable, confounder-adjusted estimates and their precision (eg, 95% confidence interval). Make clear which confounders were adjusted for and why they were included | 15 | Table 4 |
|  |  | (*b*) Report category boundaries when continuous variables were categorized |  |  |
|  |  | (*c*) If relevant, consider translating estimates of relative risk into absolute risk for a meaningful time period |  |  |

| Other analyses | 17 | Report other analyses done—eg analyses of subgroups and interactions, and sensitivity analyses | 13, 14 | Sub-analysis of clients with a follow-up VIA result |
| --- | --- | --- | --- | --- |
| **Discussion** | | | | |
| Key results | 18 | Summarise key results with reference to study objectives | 16 | First paragraph of Discussion |
| Limitations | 19 | Discuss limitations of the study, taking into account sources of potential bias or imprecision. Discuss both direction and magnitude of any potential bias | 18,19 | Discussion |
| Interpretation | 20 | Give a cautious overall interpretation of results considering objectives, limitations, multiplicity of analyses, results from similar studies, and other relevant evidence | 16 – 20 | Discussion |
| Generalisability | 21 | Discuss the generalisability (external validity) of the study results | 16 – 20 | Discussion |
| **Other information** | |  | | |
| Funding | 22 | Give the source of funding and the role of the funders for the present study and, if applicable, for the original study on which the present article is based |  | Funding statement |

**Reference**

Cuschieri S. The STROBE guidelines. Saudi J Anaesth. 2019 Apr;13(Suppl 1):S31-S34. doi: 10.4103/sja.SJA_543_18. PMID: 30930717; PMCID: PMC6398292.
