# Supplementary material for: Cervical cancer screening outcomes for HIV-positive women in the Lubombo and Manzini regions of Eswatini—Prevalence and predictors of a positive visual inspection with acetic acid (VIA) screen
Source: PLOS Glob Public Health. 2024 Apr 16;4(4):e0002760. doi: 10.1371/journal.pgph.0002760 (PMC11020862; doi:10.1371/journal.pgph.0002760)
Supplement: Attachment — Submitted filename: Response to Review Comments.docx [file pgph.0002760.s002.docx]

**Summary of Author Responses**

**Manuscript title:** Cervical Cancer Screening Outcomes for HIV-positive Women in the Lubombo and Manzini regions of Eswatini – Prevalence and Predictors of a Positive Visual Inspection with Acetic Acid (VIA) Screen

| **S/n** | **Review Comments** | **Comment** | **Response** | **Line** | **Status** |
| --- | --- | --- | --- | --- | --- |
| 1 | **Editor** | Provide direct link to base of map | <https://download.geofabrik.de/africa/swaziland.html> | 203 | Fully Addressed |
| 2 |  | Provide a link to the terms of use/license information for the base layer image or shapefile | <https://www.geofabrik.de/geofabrik/imprint.html> | 203 | Fully Addressed |
| 3 |  | In the statistical analysis, please change multivariate analysis to multivariable analysis. Also, include how the model fitness was checked and multicollinearity handled. | Multivariate has been changed to multivariable.  Assessment for model fitness and multicollinearity has been described. | 245-254, 333-337, 416-417 | Fully Addressed |
| 4 |  | Please revise the interpretation of the predictors (multivariable analysis) in a way to indicate the strength of the association (not only the direction of the association). | Thank you. This section of the results and the abstract on the predictors of a positive cervical screen have been revised. | Abstract, 316-337 | Fully Addressed |
| 5 |  | The Odds ratio in the multivariable analysis is the adjusted odds ratio (aOR), please indicate this in the table and text. | Thank you, this revision has been made. | Abstract, Table 4,  316-317 | Fully Addressed |
| 1 | **Reviewer 1** | I recommend further exploring any available data to build on the explanation of the low positivity rate, including trends over time to support the explanation that newly-introduced VIA yields lower positivity rates. | Thank you, this has been updated in the introduction | 120-130 | Fully Addressed |
| 2 |  | The authors further include that one possible explanation for differences between sites included in the study is that some personnel were better trained to perform VIA. The differences in shift patterns between medical personnel in this system and in other systems is a reasonable contributor. However, to keep this discussion point in the paper, I would recommend that the authors include clinical endpoints following screening. Better trained personnel would likely be lowering the false positivity rate of screening tests relative to a clinical endpoint of CIN2+, for example. Including clinical endpoints for at least a subset of the data would greatly strengthen this argument and the overall manuscript. | Thank you for this recommendation. However, for these clients, clinical endpoint data is not readily accessible and would require further ethical clearance. This recommendation is noted and will be incorporated in upcoming studies on cervical cancer for this cohort the team will be embarking on. We have also included this as a limitation of our study. | 413-416 | Partially Addressed |
| 3 |  | Can the authors please clarify and/or elaborate on why 95% of the women eligible for this study were included in this study? | We have revised this section and it now reads "During data extraction, the data of 23,694 women who received a cervical screen were included in the analysis."  We have also revised the first paragraph of the results to align with this. | 213-215, 268-269 | Fully Addressed |
| 4 |  | Table 2 should include statistical analysis of differences between patient characteristics across categories, in addition to the values and percentages. | Table 2 has been updated with p-values from a comparative analysis. | Table 2  289 | Fully Addressed |
| 5 |  | It would be helpful if Tables 3 and 4 included the total number of people included in each of the predictor categories. | Tables 3 and 4 have been updated to include the number of females in each category. | Tables 3 & 4 | Fully Addressed |
| 1 | **Reviewer 2** | There are missing citations in the introduction; multiple paragraphs written have only one or two citations when more are appropriate. | Thank you for this observation. The introduction has been updated. | Introduction | Fully Addressed |
| 2 |  | There is a missing discussion of the literature on prevalence and predictors of positive VIA screens in similar contexts, and within Eswatini. (For ex., what has prior research found?) | Thank you for this observation. The introduction has been updated. | Introduction | Fully Addressed |
| 3 |  | There is a missing discussion on predictors in other contexts. | Thank you for this observation. We have re-examined this, and it is available in the discussion in Lines 397-400 | 397-400 | Fully Addressed |
| 4 |  | There is a missing discussion on the rationale and reasons for completing this analysis. | This is available in the section "Cervical cancer in Eswatini" in the introduction. This section has also been updated. | 132-157 | Fully Addressed |
| 5 |  | Why were the two provinces selected? | This explanation has been provided | 159-165 | Fully Addressed |
| 6 |  | How were the health centers selected? | All health facilities in the two regions providing cervical screening services were included. | 159-165 | Fully Addressed |
| 7 |  | Line 208: “Casewise analysis was used… all patients had the outcome of interest.” This should be rewritten to indicate authors mean there were no missing observations for the outcome variable. | This revised sentence now reads, "The outcome variable had no missing observations. Cases with missing observations for the predictor variables were excluded during analysis*."* | 235-236 | Fully Addressed |
| 8 |  | I recommend re-arranging the way the results are presented for “Last VL Outcome” in a more ordered manner. (Undetectable – Suppressed – Unsuppressed – NO VL). | This has been revised. Thank you. | Table 2 | Fully Addressed |
| 9 |  | The authors would benefit from adding a short, summary paragraph of their findings before delving into the discussion. | The discussion has been revised. Thank you. | 345-351 | Fully Addressed |
| 10 |  | There is an inadequate number of citations in the discussion, with some paragraphs having zero citations despite needing them. | Thank you for this observation. We have revised the discussion and provided additional citations where appropriate. | Discussion | Fully Addressed |
